# Development and application of a rapid and visual loop-mediated isothermal amplification for the detection of *Sporisorium scitamineum* in sugarcane

**DOI:** 10.1038/srep23994

**Published:** 2016-04-01

**Authors:** Yachun Su, Yuting Yang, Qiong Peng, Dinggang Zhou, Yun Chen, Zhuqing Wang, Liping Xu, Youxiong Que

**Affiliations:** 1 Key Laboratory of Sugarcane Biology and Genetic Breeding, Fujian Agriculture and Forestry University, Ministry of Agriculture, Fuzhou 350002, China

## Abstract

Smut is a fungal disease with widespread prevalence in sugarcane planting areas. Early detection and proper identification of *Sporisorium scitamineum* are essential in smut management practices. In the present study, four specific primers targeting the core effector *Pep1* gene of *S. scitamineum* were designed. Optimal concentrations of Mg^2+^, primer and *Bst* DNA polymerase, the three important components of the loop-mediated isothermal amplification (LAMP) reaction system, were screened using a single factor experiment method and the L_16_(4^5^) orthogonal experimental design. Hence, a LAMP system suitable for detection of *S. scitamineum* was established. High specificity of the LAMP method was confirmed by the assay of *S. scitamineum*, *Fusarium moniliforme*, *Pestalotia ginkgo*, *Helminthospcrium sacchari*, *Fusarium oxysporum* and endophytes of Yacheng05-179 and ROC22. The sensitivity of the LAMP method was equal to that of the conventional PCR targeting *Pep1* gene and was 100 times higher than that of the conventional PCR assay targeting *bE* gene in *S. scitamineum*. The results suggest that this novel LAMP system has strong specificity and high sensitivity. This method not only provides technological support for the epidemic monitoring of sugarcane smut, but also provides a good case for development of similar detection technology for other plant pathogens.

Sugarcane smut, caused by the pathogen of *Sporisorium scitamineum*, was the most critical fungal disease in sugarcane industry^1^. It caused earlier germination of buds, increased the incidences of tiller, thinner stalks and black whip in tips of diseased sugarcane by infecting seed plants and jeopardizing sugarcane yield and quality^1^,^2^. Fungi-bearing seed canes and diseased ratoon canes were the initial infection sources of sugarcane smut, which was strictly prevented and controlled all over the world^1^,^3^,^4^. In Australia, an advanced sugarcane producer, a website specific to sugarcane smut established a 24-hour reporting system. In Brazil, the world’s largest sugarcane producer, as soon as one smut-infected plant appears in the segregating hybrid population in the process of sugarcane cross breeding, this combination will be eliminated. Studies on sugarcane resistance to smut began in 1990, and the resistance has become an important factor in the approval and release of a new sugarcane variety since 1995 in China. In the past ten years, the cultivar ROC22, to which no cultivar is superior regarding stability, has occupied the dominant position and has accounted for about 60–80% of the total sugarcane plantings in China. However, this variety’s insufficient resistance to sugarcane smut results in the prevalence of this disease in sugarcane producing areas. Therefore, studies on sugarcane smut are now receiving increased.

To date, some researchers have carried out studies on sugarcane resistance to smut disease with respect to cytology^5^, morphology^6^, genetics^7^ and physiological biochemistry^7^,^8^. Pathogenic detection is a fundamental work for disease control as indicated by previous research. An appropriate and early detection method for the sugarcane smut pathogen is critical in early warning, prevention and control of this disease^1^. The most common method of plant disease epidemic surveillance is to predict the prevalence of disease according to symptom investigation in the fields. This method is more reliant on visual assessment, but sometimes it is difficult to confirm infection by phenotypic symptom observation due to a latent infection of pathogens. Additionally, prevalence investigation and data collection must be carried out through the full disease epidemic period during crop season, which can last between 8 to 10 months. All these methods are costly and remain vulnerable to environmental influence^1^. Furthermore, it typically takes one week to separate and culture pathogens and conduct morphological identification^9^. Electron microscopy, on the other hand, requires expensive equipment, and an assay of serological diagnosis needs high quality antibodies^10^. In 1996, Albert and Schenck designed the amplification primer bE4/bE8 that targets the conserved sequence of the *bE* gene in *S. scitamineum* for the conventional PCR test^11^. Using the above described methods it was not possible to make timely and accurate detections of the target pathogens due to the long test period, high costs, low sensitivity and vulnerability to the influence of external environmental conditions. Our previous work designed the specific primer bEQ-F/bEQ-R and the TaqMan probe targeting the *bE* gene, and we have developed a method to detect *S. scitamineum* based on real-time quantitative fluorescence PCR^12^. By using this assay, the content of *S. scitamineum* in resistant/susceptible sugarcane genotypes could be detected, but it required expensive quantitative PCR instrument and suffered from the high cost of reagents and other consumables^12^.

Loop-mediated isothermal amplification (LAMP) is an isothermal amplification technology invented by Notomi *et al.* in 2000 which can complete automatic looping, strand displacement and DNA synthesis using *Bst* DNA polymerase and four specific primers (two inner and two outer primers)^13^. The two inner primers, which are called the forward inner primer (FIP) and the backward inner primer (BIP), contain two distinct sense and antisense sequences of the target DNA, respectively. After initiation by one of the inner primers, the following strand displacement DNA synthesis in the LAMP reaction is primed by an outer primer (either F3 or B3). A released single-stranded DNA serves as a template for DNA synthesis facilitated by the second inner and outer primers that hybridize to the other end of the target. This results in a DNA sequence which can form a stem-loop structure at both ends. This stem-loop DNA is the starting material for the subsequent LAMP auto-cycling amplification. An accumulation of 10^9^ copies of target DNA are observed in less than 1 h. The amplification products are stem-loop DNAs with several inverted repeats of the target and cauliflower-like structures with multiple loops^13^. The LAMP assay has high amplification rates, and is time efficient, easy to manipulate and results in visible products^13^. It does not rely on expensive instruments or temperature gradients. These advantages make LAMP a good onsite rapid detection method and it has been widely used to detect viruses, bacteria and fungi^14^,^15^,^16^,^17^. Nie designed four primers specific to the coat protein gene in *Potato virus* Y (PVY) and established a LAMP system, a one-step RT-LAMP and a two-step RT-LAMP system for the detection of PVY^14^. The results showed that 234 of 240 samples were accurately determined^14^. Nagdev *et al.* designed primers according to the sequence of the IS6110 gene of *Mycobacterium tuberculosis*, and found that the sensitivity and specificity of the optimized LAMP systems were 88.23% and 80%, respectively, which was higher than that of nested PCR (52.9% sensitivity)^15^. There have also been some reports on the application of LAMP technology in the detection of sugarcane pathogenic bacteria and transgenic lines^18^,^19^,^20^,^21^,^22^. Liu *et al.* and Ghai *et al.* established the LAMP method to detect *Leifsonia xyli* subsp. *xyli*, where Liu *et al.* found that the sensitivity of LAMP was 10 times than that of conventional PCR^18^,^19^. Ghai *et al.* reported specific LAMP primers that could detect up to 3 pg/μL of genomic bacterial DNA and were as sensitive as the enzyme-linked immuno sorbent assay (ELISA) but much quicker^19^. Keizerweerd *et al.* established the RT-LAMP method for the detection of *Sugarcane mosaic virus* and *Sorghum mosaic virus*^20^. Chandra *et al.* developed a LAMP assay to detect *Colletotrichum falcatum* in sugarcane, with sensitivity 10 times higher than that of conventional PCR^21^. Zhou *et al.* optimized a LAMP system to detect *cry1Ac* transgenic sugarcane, with a sensitivity of 10–100 times higher than that of conventional PCR^22^. However, there is still no report on using LAMP to detect *S. scitamineum* in sugarcane.

Doehlemann *et al.* indicated that the protein essential during penetration 1 (Pep1) of *Ustilago maydis* was a secreted effector required for its successful invasion into plant cells, concluding that the conservative feature of Pep1 has a conserved function essential for establishing compatibility that was not restricted to the maize-*U. maydis* interaction^23^. Hemetsberger *et al.* showed that Pep1 could suppress plant immunity by inhibiting plant peroxidase, which served as an effective inhibitor for early defense reactions in plants^24^. Hemetsberger *et al.* also revealed that Pep1 was a core fungal effector in monocot and dicot smut diseases, with a highly conserved sequence^25^. Hence, in this study, specific primers were designed to target the *Pep1* gene of *S. scitamineum* (gene ID: SmutADNA4_GLEAN_10005329_Smut) identified by homology in the *S. scitamineum* whole genome sequence obtained in our previous research^26^. The single factor experiment combined with orthogonal experimental design was conducted to optimize the three impact factors of the LAMP reaction system (Mg^2+^ concentration, primer ratio and *Bst* DNA polymerase content) for *S. scitamineum* detection. The sensitivity and specificity of the developed method were also verified. In addition, the accuracy was verified by determining *S. scitamineum* in artificially inoculated sugarcane. This study aims to establish a LAMP system for rapid detection of *S. scitamineum* in sugarcane before symptoms of smut disease appear, laying a foundation for epidemiological studies on sugarcane, disease control and exit–entry quarantine in the exchange of sugarcane germplasms.

## Results

### Single factor experiment of LAMP

**Figure 1:**
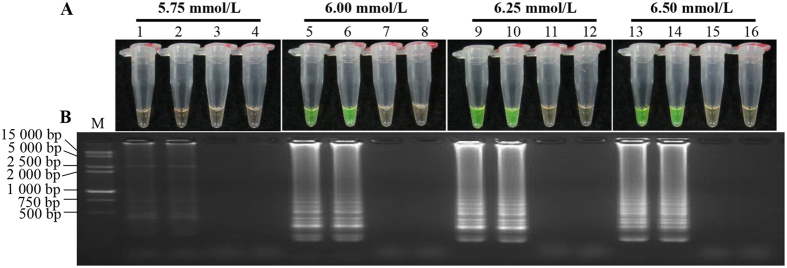
Optimization of Mg^2+^ concentration in the LAMP reaction. (**A**) LAMP products detected by 1 000×SYBR Green I (Biotek Co., Ltd., Beijing, China). (**B**) Detection of LAMP products by agarose gel electrophoresis stained by EB (ethidium bromide). Tubes and lanes 3, 7, 11 and 15: negative control. Tubes and lanes 4, 8, 12 and 16: blank control. Tubes and lanes 1 and 2, 5 and 6, 9 and 10, 13 and 14: the positive plasmid pMD19-T-Pep1, repeated twice. Tubes and lanes 1–4, 5–8, 9–12 and 13–16: concentrations of Mg^2+^ were 5.75 mmol/L, 6.00 mmol/L, 6.25 mmol/L and 6.50 mmol/L, respectively. Lane M: DL 15 000 + 2 000 DNA marker.

**Figure 2:**
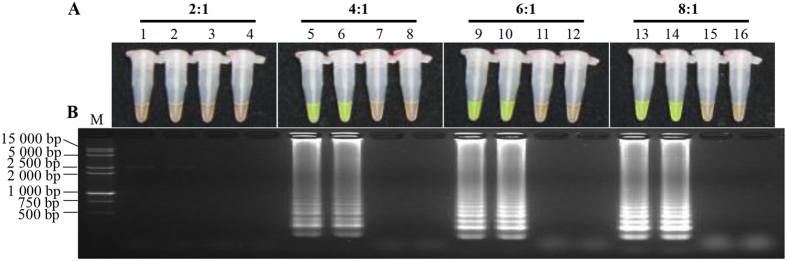
Optimization of ratios of inner and outer primers in the LAMP reaction. (**A**) LAMP products detected by 1 000×SYBR Green I (Biotek Co., Ltd., Beijing, China). (**B**) Detection of LAMP products by agarose gel electrophoresis stained by EB (ethidium bromide). Tubes and lanes 3, 7, 11 and 15: negative control. Tubes and lanes 4, 8, 12 and 16: blank control. Tubes and lanes 1 and 2, 5 and 6, 9 and 10, 13 and 14: the positive plasmid pMD19-T-Pep1, repeated twice. Tubes and lanes 1–4, 5–8, 9–12 and 13–16: ratios of inner and outer primers were 2:1, 4:1, 6:1 and 8:1, respectively. Lane M: DL 15 000 + 2 000 DNA marker.

**Figure 3:**
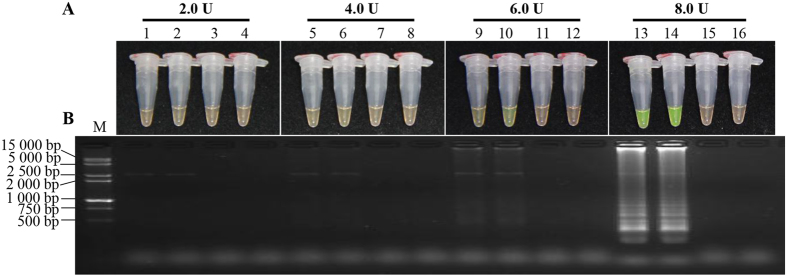
Optimization of concentration of *Bst* DNA polymerase in the LAMP reaction. (**A**) LAMP products detected by 1 000×SYBR Green I (Biotek Co., Ltd., Beijing, China). (**B**) Detection of LAMP products by agarose gel electrophoresis stained by EB (ethidium bromide). Tubes and lanes 3, 7, 11 and 15: negative control. Tubes and lanes 4, 8, 12 and 16: blank control. Tubes and lanes 1 and 2, 5 and 6, 9 and 10, 13 and 14: the positive plasmid pMD19-T-Pep1, repeated twice. Tubes and lanes 1–4, 5–8, 9–12 and 13–16: *Bst* DNA polymerase concentrations were 2.0 U, 4.0 U, 6.0 U and 8.0 U, respectively. Lane M: DL 15 000 + 2 000 DNA marker.

In order to obtain a LAMP system suitable to detect *S. scitamineum*, a single factor optimization experiment was conducted on a Mg^2+^ concentration (5.75 mmol/L, 6.00 mmol/L, 6.25 mmol/L and 6.50 mmol/L), an inner *vs.* outer primer ratio (2:1, 4:1, 6:1 and 8:1) and the *Bst* DNA polymerase content (2.0 U, 4.0 U, 6.0 U and 8.0 U) in the LAMP system (Figs 1, 2, 3). Appropriate concentration ranges were screened. Combining tests of SYBR Green I method and product electrophoresis, we found that with the optimal conditions of Mg^2+^ (5.75 mmol/L, 6.00 mmol/L, 6.25 mmol/L and 6.50 mmol/L) (Fig. 1), ratios of inner *vs.* outer primer (4:1, 6:1 and 8:1) (Fig. 2) and *Bst* DNA polymerase (4.0 U, 6.0 U and 8.0 U) (Fig. 3), flavogreen fluorescent color and amplification bands were displayed. However, in the negative and blank controls, the tubes maintained the orange color in the amplification products of the SYBR Green I test and no band was detected in electrophoresis.

### Orthogonal experiment of LAMP

**Figure 4:**
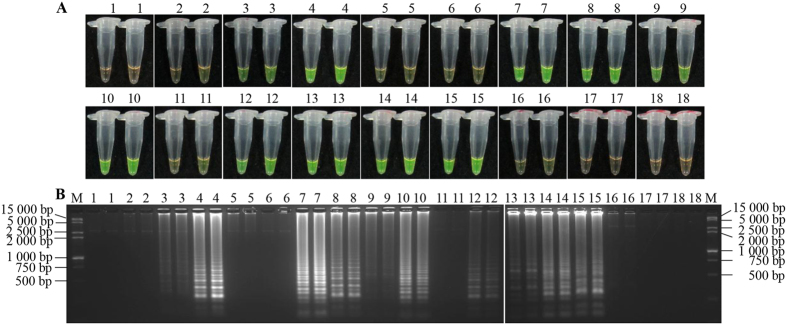
The detection results based on different reaction systems according to orthogonal test. (**A**) LAMP products detected by 1 000×SYBR Green I (Biotek Co., Ltd., Beijing, China). (**B**) Detection of LAMP products by agarose gel electrophoresis stained by EB (ethidium bromide). Tubes and lanes 1–16 are shown in Table 1. Tube and lane 17: negative control. Tube and lane 18: blank control. Lane M: DL 15 000 + 2 000 DNA marker.

Subsequent reactions were performed under 16 combinations in a LAMP orthogonal design experiment (Table 1). Color changes were observed in the amplification when concentrations of Mg^2+^, inner and outer primers and *Bst* DNA polymerase were altered. As shown in Fig. 4A, combinations 1, 2, 5, 6, 11 and 16 exhibited no or extremely weak flavogreen fluorescent signals in the SYBR Green I test. Additionally, there were few or no amplification band(s) displayed after electrophoretic analysis (Fig. 4B). LAMP products of combination 7 showed high flavogreen fluorescence, and displayed bright typical ladder-like bands after electrophoresis, apparent in distinction from the negative and blank controls, whereas combinations 4, 8 and 10 showed intensive flavogreen fluorescent signals but weaker band brightness than combination 7 in electrophoresis. For other combinations, results were not as good as combination 7. Thus, the LAMP reaction conditions in combination 7 were optimal for detecting *S. scitamineum* in sugarcane, which consisted of Mg^2+^ at a concentration of 6.00 mmol/L, a ratio of inner *vs.* outer primers of 6:1, and a concentration of *Bst* DNA polymerase of 8.0 U. The optimized LAMP reaction conditions including 4 mmol/L MgSO_4_ (50 mmol/L), 1.4 mmol/L dNTPs (10 mmol/L), 2.5 μL 10×ThermoPol Reaction Buffer [containing 20 mmol/L Tris-HCl pH 8.8, 10 mmol/L KCl, 2 mmol/L MgSO_4_, 10 mmol/L (NH_4_)_2_SO_4_, 0.1% Triton X-100, NEB company], 1.2 μmol/L each of FIP and BIP primers, 0.2 μmol/L each of F3 and B3 primers, 8.0 U *Bst* DNA polymerase (8 000 U/mL, NEB company), DNA template 1.0 μL, ddH_2_O were complemented to a volume of 25 μL.

### Specificity of LAMP

**Figure 5:**
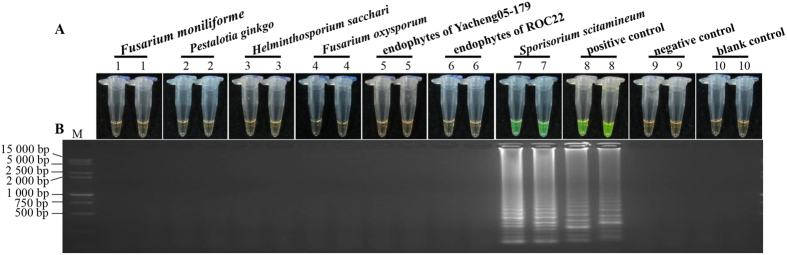
The detection results of the LAMP reaction specificity. (**A**) LAMP products detected by 1 000×SYBR Green I (Biotek Co., Ltd., Beijing, China). (**B**) Detection of LAMP products by agarose gel electrophoresis stained by EB (ethidium bromide). Tubes and lanes 1–7: Genomic DNA of *Fusarium moniliforme*, *Pestalotia ginkgo*, *Helminthosporium sacchari*, *Fusarium oxysporum*, the endophytes of sugarcane genotypes Yacheng05–179 (smut resistance) and ROC22 (smut susceptible), *Sporisorium scitamineum*, respectively. Tube and lane 8: the positive plasmid pMD19-T-Pep1. Tube and lane 9: negative control. Tube and lane 10: blank control. Lane M: DL 15 000 + 2 000 DNA marker.

To know whether the optimized LAMP system was specific, the common sugarcane pathogenic DNA was tested. Only target DNA of *S. scitamineum* and pMD19-T-Pep1 plasmid exhibited positive reactions, while no positive reaction was observed in the other six commonly encountered sugarcane pathogens, i.e. *Fusarium moniliforme*, *Pestalotia ginkgo*, *Helminthospcrium sacchari*, *Fusarium oxysporum*, and two endophytes from Yacheng05-179 and ROC22 (Fig. 5), indicating the newly developed method has good specificity for detecting *S. scitamineum*.

### Comparison of the sensitivity of LAMP and conventional PCR assay

**Figure 6:**
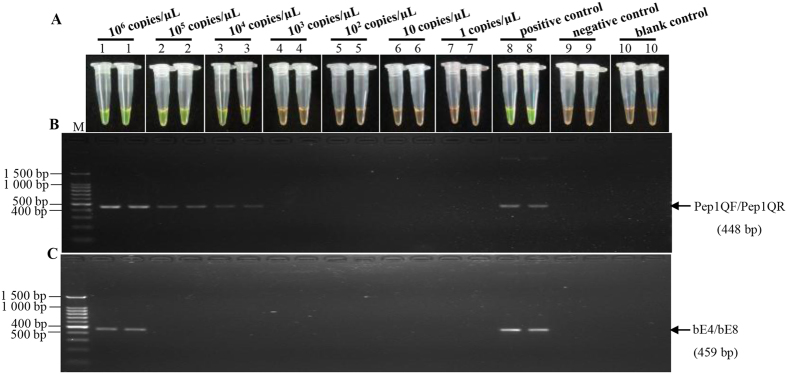
Sensitivity comparison of the LAMP and conventional PCR assay using the genomic DNA of *Sporisorium scitamineum* as template. (**A**) LAMP products detected by 1 000×SYBR Green I (Biotek Co., Ltd., Beijing, China). (**B**) Conventional PCR products of *Pep1* gene detected by agarose gel electrophoresis stained by EB (ethidium bromide). (**C**) Conventional PCR products of *bE* gene detected by agarose gel electrophoresis stained by EB (ethidium bromide). Tubes and lanes 1–7: ten-fold serial dilutions (10^6^–1 copies/μL) of the genomic DNA of *Sporisorium scitamineum*, respectively. Tube and lane 8: positive control. Tube and lane 9: negative control. Tube and lane 10: blank control. Lane M: DL 100 bp DNA marker.

The LAMP assay showed excellent results with regard to sensitivity, compared to conventional PCR by conducting amplification using ten-fold diluted samples of *S. scitamineum* genomic DNA (10^6^ copies/μL–1 copies/μL) as a template. As indicated by the color transition of LAMP reaction products (Fig. 6A), the lowest limit of *S. scitamineum* detection was 10^4^ copies/μL for LAMP, whereas in conventional PCR for *Pep1* (Fig. 6B) and *bE* tests (Fig. 6C), the detection limits were 10^4^ copies/μL and 10^6^ copies/μL, respectively. This implied that the LAMP assay had a wider dynamic range and was nearly 100 times more sensitive than conventional PCR for detection of *bE* of *S. scitamineum*^11^.

### Detection of *S. scitamineum* in sugarcane inoculated with smut pathogen

**Figure 7:**
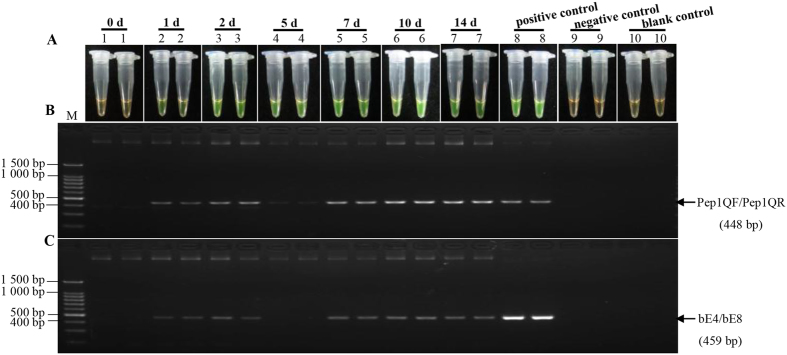
The detection results of the LAMP and conventional PCR on sugarcane ROC22 buds challenged with *Sporisorium scitamineum.* (**A**) LAMP products detected by 1 000×SYBR Green I (Biotek Co., Ltd., Beijing, China). (**B**) Conventional PCR products of *Pep1* gene detected by agarose gel electrophoresis stained by EB (ethidium bromide). (**C**) Conventional PCR products of *bE* gene detected by agarose gel electrophoresis stained by EB (ethidium bromide). Tubes and lanes 1–7: the samples of sugarcane ROC22 buds challenged with *Sporisorium scitamineum* at 0 d, 1 d, 2 d, 5 d, 7 d, 10 d and 14 d, respectively. Tube and lane 8: positive control. Tube and lane 9: negative control. Tube and lane 10: blank control. Lane M: DL 100 bp DNA marker.

In order to test the reliability of the LAMP reaction system optimized in this study, we inoculated buds of ROC22 with a spore suspension of *S. scitamineum*. At 0 d, 1 d, 2 d, 5 d, 7 d, 10 d and 14 d, genomic DNA of the inoculated buds was isolated and tested by the developed LAMP assay and a conventional PCR. A total of six sugarcane samples after inoculation with *S. scitamineum* were detected by the LAMP method developed in this study, and the conventional PCR assay targeting *bE* gene and *Pep1* gene. No amplification was observed in the mock (0 h), negative and blank controls (Fig. 7). Positive reactions were displayed in all the infected samples, as was the case in the positive controls by LAMP assay (Fig. 7A) and conventional PCR assay for the *Pep1* gene in *S. scitamineum* (Fig. 7B), indicating a positive detection rate of 100% for genomic DNA of cane buds incubated for 1 d to 14 d after inoculation. In the conventional PCR reaction for the *bE* gene (Fig. 7C), no visible band could be observed at 5 d, suggesting a positive detection rate of 83.33%.

## Discussion

Sugarcane smut is one of the most prevalent diseases in sugarcane industry worldwide. High sensitivity and efficiency in the detection of *S. scitamineum* infection is desirable in the process of sugarcane smut resistance breeding. Currently, the common methods for *S. scitamineum* detection involve field disease symptom investigation, electron microscopic observation, and serologic diagnosis, which are complicated to manipulate. In addition, accuracy and sensitivity vary greatly among different approaches^1^,^10^. Albert and Schenck designed the primers bE4 and bE8 targeting the *bE* gene for detection of *S. scitamineum* by conventional PCR^11^. In our previous study^12^, a TaqMan real-time PCR assay for detection and quantification of *S. scitamineum,* which allowed detection of the target pathogen in the infected sugarcane plants, was established. However, this method requires special equipment, including a PCR device and a real-time quantitative fluorescence PCR device, which raises the instrumental and time costs of pathogen detection. By LAMP, the reaction can be carried out in a PCR tube incubated at one certain temperature for nearly 1 h^13^. In addition, the operation of opening the tube cover is not required because inspection of the detection results due to a visual color change makes it possible to complete the target gene amplification and product detection in one step, which reduces the danger of cross contamination^22^.

For efficient amplification, smart design and appropriate dosage of primers in a LAMP system can ensure the loop hairpin structure in the initial and the continuous amplifications in the reaction^13^,^27^. It could also promote the specific amplification that is mediated by the chain replacement enzyme (*Bst* DNA polymerase) under constant temperature. Additionally, primer quality and inner *vs.* outer primer ratio play key roles in the LAMP reaction system as these factors have more significant influence on its effectiveness, repeatability and sensitivity^13^,^28^. As reported, the concentration of inner primers (FIP and BIP) was often higher (4–10 times) than that of outer primers (F3 and B3)^14^,^29^. In this study, four primers (Pep1F3/Pep1B3 and Pep1FIP/Pep1BIP) corresponding to six independent segments targeting the *Pep1* gene in *S. scitamineum* were designed. An optimal dosage ratio (6:1) of inner *vs.* outer primer (Figs 2 and 4) distinguished the negative and positive controls.

As *Bst* DNA polymerase is a Mg^2+^ dependent enzyme, the Mg^2+^ concentration was assumed to be the most important component in the LAMP assay^30^. Mg^2+^ also binds to dNTPs, primers and templates, affecting specificity of the LAMP reaction, amplification of product yield and the formation of primer dimers^14^,^28^. Liu *et al.* reported that the excessive concentration of Mg^2+^ led to incorrect binding between primer and template, causing a decreased specificity of the LAMP reaction^18^. Chandra *et al.* also found that higher concentrations of Mg^2+^, those which exceed the optimum level, might result in a pseudo-positive result even in the negative sample^21^. Although the MgCl_2_ reagent was also used in some investigations^14^,^29^, the MgSO_4_ reagent was recommended for the 10×ThermoPol Reaction Buffer supplied in the *Bst* DNA polymerase kit mentioned in our experiments. Here, when the Mg^2+^ concentration ranged from 5.75 mmol/L to 6.50 mmol/L (Fig. 1), LAMP results showed a distinction between positive and negative controls. As suggested by electrophoresis, a Mg^2+^ concentration of 6.25 mmol/L was optimal (Fig. 1B). This was consistent with the results reported by Nie and Liu *et al.*, which suggested the existence of an optimum concentration of Mg^2+^ during the LAMP reaction^14^,^29^. Fluoroscopic and electrophoretic signals were extremely weak at lower dosages of the *Bst* DNA polymerase (2.0 U) (Fig. 3). When the concentration of the *Bst* DNA polymerase was 8.0 U, negative and positive controls could clearly be distinguished. This was similar to the Nie’s findings that showed that the product concentration was reduced due to a higher consumption rate of the *Bst* DNA polymerase^14^.

Normally, a single factor experiment requires firstly to define initial reaction conditions according to experiences, then one factor is altered to screen approximately optimal concentration ranges under the condition that all the other factors remain unchanged. This method requires more lab work and higher costs. Meanwhile, it is difficult to truly achieve the optimal experimental results using this method due to possible interactions between the factors not considered in the reaction system^12^. Orthogonal experimental design considers the interaction effects between the reaction factors, with the advantages of scattered balance and comprehensive comparisons^31^. Therefore, the optimal combination can be rapidly established. However, in order to know the appropriate dosage ranges of impact factors before employing orthogonal experimental design, researchers usually adjust concentration levels of factors to obtain the optimal reaction system^32^. In the present study, a double optimized LAMP reaction system was rapidly developed that was suitable to detect *S. scitamineum* by employing a single factor experiment followed by an orthogonal experimental design.

Several studies found that the specificity and sensitivity of the LAMP technology were higher than those of conventional PCR and RT-PCR^21^,^33^,^34^. Guo *et al.* found that the sensitivity of LAMP was 10 times higher than that of the conventional PCR for detecting *Mycoplasma hyopneumoinae*^33^. Similarly, Wang *et al.* indicated that the LAMP method was 10 times more sensitive than that of conventional PCR for the detection of the *cry1Ac* gene in *Bt* transgenic *Oryza sativa*^34^. Chandra *et al.* demonstrated that the sensitivity of the LAMP assay was 5 times greater than that of conventional PCR for detection of gDNA from *C. falcatum*-infected sugarcane tissues^21^. In this study, except for the positive control, LAMP had no cross reaction with other pathogens, which suggests its high specificity. Sensitivity analysis showed that the detection limit of the LAMP assay was 100 times higher than that of conventional PCR targeting the *bE* gene for *S. scitamineum*. In addition, the LAMP reaction was positive for all tested cane buds that were artificially inoculated with *S. scitamineum*.

In conclusion, the present work established a LAMP method with the specific primers of Pep1F3/Pep1B3 and Pep1FIP/Pep1BIP for *S. scitamineum* in sugarcane. Double optimal methods, the single factor experiment as well as L_16_(4^5^) orthogonal experimental designs, were employed to optimize the LAMP reaction system. This novel LAMP assay was sensitive and rapid in the detection of *S. scitamineum*. It might be also suitable to detect imported or exported sugarcane seeds or seed stems, and provide technical support for realizing smut-free sugarcane supervision and administration.

## Methods

### Plant materials and treatments

Both the *S. scitamineum* spores and sugarcane genotype ROC22 were from the Key Laboratory of Sugarcane Biology and Genetic Breeding, Ministry of Agriculture (Fuzhou, China). Smut whips in diseased plants of ROC22 collected in the fields were sealed and stored in 4 °C refrigerator after dried under sunshine or in an incubator at 35 °C. Spore suspension was prepared by diluting smut spores with ddH_2_O. Then, 100 μL of diluent was applied on a potato dextrose agar (PDA) plate containing 75 μg/mL of streptomycin and cultured at 28 °C in darkness. After 4 d, the single spore was re-cultured on a new PDA plate at 28 °C in darkness for 5 d. Finally, it was used to extract hypha DNA by the SDS (Sodium Dodecyl Sulfonate) method^35^. Hypha DNA was treated by 100 μg/mL RNase A (Promega company, USA) and its purity was determined by electrophoresis and spectrophotometer, and then stored at −20 °C.

Healthy and mature sugarcane stalks of ROC22 with uniform growth rates were selected for the test. They were cut into single-bud segments and soaked in flowing water for 24 h, then placed in incubators at 32 °C with 65% humidity and 24 h lighting to accelerate germination. When the cane buds were 2 cm long, a 0.5 μL microliter syringe was used to conduct inoculation on cane buds with a 5 × 10^6^/mL of *S. scitamineum* spore suspension. The 16 h of light/8 h of darkness alternative culture was performed for the treated materials at 28 °C, from which 6 cane buds were selected at 0 d, 1 d, 2 d, 5 d, 7 d, 10 d and 14 d for the genome DNA extraction by CTAB (hexadecyl trimethyl ammonium bromide) method^35^.

### Primer design and LAMP reaction

According to the sequences of *Pep1* (gene ID: Smut ADNA4 _GLEAN_ 10005329_Smut) in *S. scitamineum*^26^, a pair of clone primers Pep1F (5′-CACTGACTCAAGCCATCCTGC-3′) and Pep1R (5′-AGTTGCCGAGACCGCTGA-3′) were designed using Primer Premier 5.0 software. PCR amplification was performed using the *S. scitamineum* genome DNA as a template. The PCR reaction system including 2.0 μL dNTP (2.5 mmol/L), 2.5 μL 10×*Ex* Taq Buffer (Mg^2+^ Plus), 0.125 μL *Ex* Taq enzyme (5 U/μL), 1.0 μL Pep1F (10 μmol/L) and 1.0 μL Pep1R (10 μmol/L), 1.0 μL template DNA (25 ng/μL), and ddH_2_O was complemented to a final volume of 25 μL. The PCR amplification procedure was: 94 °C initial denaturation for 4 min; 94 °C denaturation for 30 s, 52 °C annealed for 30 s, 72 °C extension for 30 s, cycled 35 times; 72 °C re-extension for 10 min. After purification by gel extraction, all PCR amplification products (507 bp) were built onto a pMD19-T Cloning Vector (Takara, China) and transformed into *Escheria coli* DH5α. Positive clones were sequenced by Shanghai Sangon Biotech Co., Ltd. (China). The plasmid, named pMD19-T-Pep1, was stored for future use.

**Figure 8:**
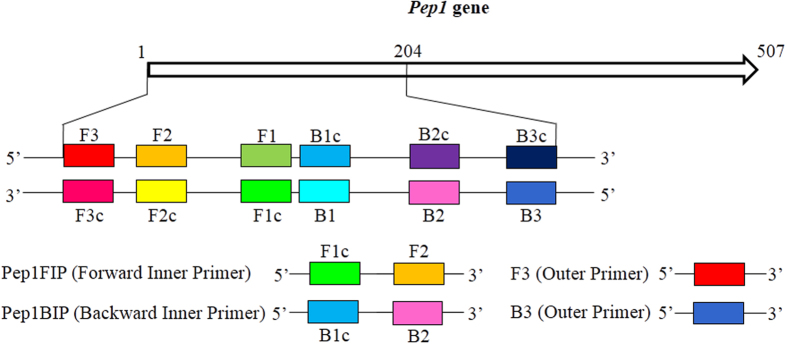
Schematic representation of each LAMP primer targeting *Pep1* gene in *Sporisorium scitamineum.* Construction of the inner primers Pep1FIP and Pep1BIP were shown. F1c and B1c were complementary to F1 and B1, respectively.

Based on *Pep1* sequences, software PrimerExplorer 4.0 (http://primerexplorer.jp/e/) was used to design the LAMP primers, from which four primers, two outer primers Pep1F3 and Pep1B3 and two inner primers Pep1FIP (F1c + F2) and Pep1BIP (B1c + B2) (Fig. 8) were screened. These primers were synthesized by Shanghai Sangon Biotech Co., Ltd. (China). The Pep1FIP and Pep1BIP primers were at HPLC (high performance liquid chromatography) grade.

Pep1F3: 5′-CACTGACTCAAGCCATCCTG-3′;

Pep1B3: 5′-GAAGCGCTCCCTTTACGC-3′;

Pep1FIP(F1c + F2): 5′-AATCGGGCAGCGGTATAGCG-GCTAGCCTTCGTGCTCAC-3′;

Pep1BIP(B1c + B2): 5′-CAAGCTGGTGCAATTCCCGATG-CTGTAGCACACACCGAGTTC-3′.

### Optimization of LAMP reaction conditions

The LAMP reaction was carried out in a 25 μL mixture containing 3.75 mmol/L MgSO_4_ (50 mmol/L), 1.4 mmol/L dNTPs (10 mmol/L), 2.5 μL 10×ThermoPol Reaction Buffer [including 20 mmol/L Tris-HCl pH 8.8, 10 mmol/L KCl, 2 mmol/L MgSO_4_, 10 mmol/L (NH_4_)_2_SO_4_ and 0.1% Triton X-100, NEB company], 0.8 μmol/L each of FIP and BIP primers, 0.2 μmol/L each of F3 and B3 primers, 8.0 U *Bst* DNA polymerase (8 000 U/mL, NEB company), 1.0 μL template DNA, and ddH_2_O, which was complemented to a volume of 25 μL. The blank control (ddH_2_O), negative control (FN40 tissue culture seedlings free of fungal pathogens) and positive control (pMD19-T-Pep1 plasmid) were set for the experiment. Key compositions of the LAMP system were optimized using a single factor experiment and an orthogonal experiment to obtain an optimal LAMP system which is stable and sensitive enough for the detection of *S. scitamineum*.

### Single factor experiment

During the first optimization test, a single factor experiment with three factors and four levels was conducted to screen the dosage of the major factors (Mg^2+^ concentration, inner *vs.* outer primer ratio and *Bst* DNA polymerase content) influencing the LAMP reaction. Mg^2+^ concentrations were set to 5.75 mmol/L, 6.00 mmol/L, 6.25 mmol/L and 6.50 mmol/L, respectively. For the fixed outer primer concentration of 0.2 μmol/L, the inner *vs.* outer primer ratios were set to 2:1, 4:1, 6:1 and 8:1, respectively. Four different *Bst* DNA polymerase concentrations, 2.0 U, 4.0 U, 6.0 U and 8.0 U, were tested. The amplification procedure followed 65 °C incubation for 1 h, and a 80 °C hot bath for 5 min to terminate the reaction. The products were detected by color transition and 2.0% agarose gel electrophoresis. 1.5 μL 1 000×SYBR Green I fluorescent dyes (Biotek Co., Ltd., Beijing, China) were added and dropped inside the tube cap before reaction, which allowed the positive product to be indicated by color transition from orange to flavogreen after incubation; those showing intense ladder-like electrophoretic bands under UV light were defined as positive. All tests were repeated twice.

### Orthogonal experiment

Due to interactions between factors of LAMP reaction, L_16_(4^5^) orthogonal experimental design was selected for this study^31^. It was carried out on the basis of the single factor experiment to conduct orthogonal screening with three factors and four levels (Table 1). Each combination was repeated twice.

### Specificity of LAMP

Specificity of primers was verified by the optimized LAMP system using 100 ng of the following common sugarcane pathogenic DNA as template, including *F. moniliforme*, *P. ginkgo*, *H. sacchari*, *F. oxysporum* and *S. scitamineum* genome DNA. In addition, cane exudates from smut resistant and susceptible sugarcane genotypes (Yacheng05–179 and ROC22) were cultivated at PDA liquid medium (containing 75 μg/mL streptomycin) at 28 °C for 1 d to procure the endophytes. Genome DNA of these pathogens and endophytes was extracted following SDS protocol established by Que *et al.*^35^. The amplification products were detected by color transition and 2.0% agarose gel electrophoresis. The experiment was repeated twice.

### Analytical sensitivity of LAMP

According to the formulas of MW = genome length × 660 dalton/bp and copies/mL = 6.02 × 10 23 × (concentration g/mL)/(MW g/mol)^36^, the copy number of *S. scitamineum* (19.8 Mb)^26^ was calculated. A ten-fold gradient dilution was conducted on *S. scitamineum* genome DNA (217 ng/μL, amount to 10^7^ copies/μL) to obtain a total of seven samples which ranged from 10^6^ copies/μL–1 copies/μL. The optimized LAMP amplification was conducted and repeated twice.

Additionally, conventional PCR amplification was conducted using *S. scitamineum* testing primers of bE4 (5′-CGCTCTGGTTCATCAACG-3′) and bE8 (5′-TGCTGTCGATGGAAGGTGT-3′) for the *bE* gene^11^, as well as another pair of primers Pep1QF (5′-TAGCCTTCGTGCTCACCTTTACC-3′) and Pep1QR (5′-TGCTTTCCGCCATCCTCCT-3′) which were designed by ourselves based on the *Pep1* gene sequences. The conventional PCR reaction system containing 2.0 μL dNTP (2.5 mmol/L), 2.5 μL 10×*Ex* Taq Buffer (Mg^2+^ Plus), 0.125 μL *Ex* Taq enzyme (5 U/μL), 1.0 μL forward primer (10 μmol/L) and 1.0 μL reward primer (10 μmol/L), 1.0 μL template DNA, ddH_2_O was complemented to a volume of 25 μL. The PCR amplification was performed under the following conditions: 94 °C initial denaturation for 4 min; 94 °C denaturation for 30 s, 55 °C (*bE* gene) or 60 °C (*Pep1* gene) annealed for 30 s, 72 °C extension for 30 s, cycled for 35 times; 72 °C re-extension for 10 min^11^. The 2.0% agarose gel electrophoresis was used to test the PCR amplified product.

### Evaluation of LAMP using *S. scitamineum* inoculated samples

Two-bud sets of ROC22 were inoculated with 0.5 μL of the *S. scitamineum* suspension containing 5 × 10^6^ spores/mL in 0.01% (v/v) Tween-20. All the treated samples were planted in sterile soil and cultivated at 28 °C in 16 h of light and 8 h of darkness. At each time point of 0 d, 1 d, 2 d, 5 d, 7 d, 10 d and 14 d, five inoculated buds were collected for DNA extraction using the CTAB method. These DNA samples (1 μg) were subjected to the optimized LAMP reaction and conventional PCR (*bE* gene and *Pep1* gene detection) reaction systems. The 2.0% agarose gel electrophoresis was used to detect the amplified product of the conventional PCR.

## Additional Information

**How to cite this article**: Su, Y. *et al.* Development and application of a rapid and visual loop-mediated isothermal amplification for the detection of *Sporisorium scitamineum* in sugarcane. *Sci. Rep.* **6**, 23994; doi: 10.1038/srep23994 (2016).

## Figures and Tables

**Table 1:** [L_16_(4^5^)] orthogonal design for LAMP system.

Horizontal combination	Factor		
	Mg^2+^ concentration (mmol/L)	inner *vs.* outer primer ratios	*Bst* DNA polymerase concentration (U)
1	5.75	2:1	2.0
2	5.75	4:1	4.0
3	5.75	6:1	6.0
4	5.75	8:1	8.0
5	6.00	2:1	4.0
6	6.00	4:1	2.0
7	6.00	6:1	8.0
8	6.00	8:1	6.0
9	6.25	2:1	6.0
10	6.25	4:1	8.0
11	6.25	6:1	2.0
12	6.25	8:1	4.0
13	6.50	2:1	8.0
14	6.50	4:1	6.0
15	6.50	6:1	4.0
16	6.50	8:1	2.0
